# Adaptation of endoscopic submucosal dissection techniques for endoscopic full-thickness resection: a review of key steps and technical aspects

**DOI:** 10.1016/j.vgie.2023.09.015

**Published:** 2023-10-05

**Authors:** James Weiquan Li, Noriya Uedo, Satoki Shichijo

**Affiliations:** 1 Department of Gastrointestinal Oncology, Osaka International Cancer Institute, Osaka, Japan; 2 Department of Gastroenterology and Hepatology, Changi General Hospital, Singapore Health Services, Singapore

## Abstract

**Background and Aims:**

GI stromal tumors (GISTs) represent the most common mesenchymal tumors of the GI tract. Guidelines recommend the removal of histologically proven gastric GISTs >2 cm. While the conventional treatment of a gastric GIST involves surgical excision, endoscopic full-thickness resection (EFTR) has been described as an acceptable alternative. We aim to outline how the key steps used in endoscopic submucosal dissection (ESD) can be adapted to the performance of exposed EFTR and discuss the variations in technical aspects between the 2 procedures.

**Methods:**

We use a video case illustration with a comprehensive narrative to highlight the similarities and differences in equipment used and techniques in EFTR and ESD. Images and graphical illustrations are also used to describe these techniques.

**Results:**

ESD techniques and equipment can be adapted for use in EFTR of gastric GISTs. Principles such as deep mucosal incision, the appropriate use of traction, and identification of vessels for prophylactic coagulation help to ensure a safe and efficient procedure. The main difference in EFTR is the need for general anesthesia, starting the mucosal incision as close to the tumor margin as possible, submucosal dissection around the surface of the tumor capsule, and a strong closure method for the muscle defect.

**Conclusions:**

The equipment and techniques in ESD can be adapted to EFTR for gastric GISTs by endoscopists who are familiar with ESD techniques.

## Background and Aims

GI stromal tumors (GISTs) represent the most common mesenchymal tumors of the GI tract. The European Society for Medical Oncology, the European Reference Network for Rare Adult Solid Cancers, the European Reference Network for Genetic Tumor Risk Syndromes,^1^ and the Japanese Society of Clinical Oncology,^2^ as well as the recent European Society of Gastrointestinal Endoscopy guidelines,^3^ recommend the removal of histologically proven gastric GISTs. This is because the true malignant potential of a GIST can only be determined after resection.^4^^,^^5^ While active surveillance is an option in lesions smaller than 20 mm,^6^ this places a burden of repeat follow-up gastroscopies with EUS in young patients. Moreover, compliance to surveillance recommendations is poor,^7^ which may be because an optimal and evidence-based protocol for such active surveillance is lacking.

Conventional treatment of gastric GISTs involves surgical excision.^1^ However, endoscopic full-thickness resection (EFTR) has been described as an acceptable alternative if complete excision can be achieved without tumor rupture.^1^^,^^8^^,^^9^ This video aims to outline how the key steps used in endoscopic submucosal dissection (ESD) can be adapted to EFTR and discusses the variations in technical aspects between the 2 procedures. A case presentation will be used for illustration of the procedure.

## Case Presentation

**Figure 1:**
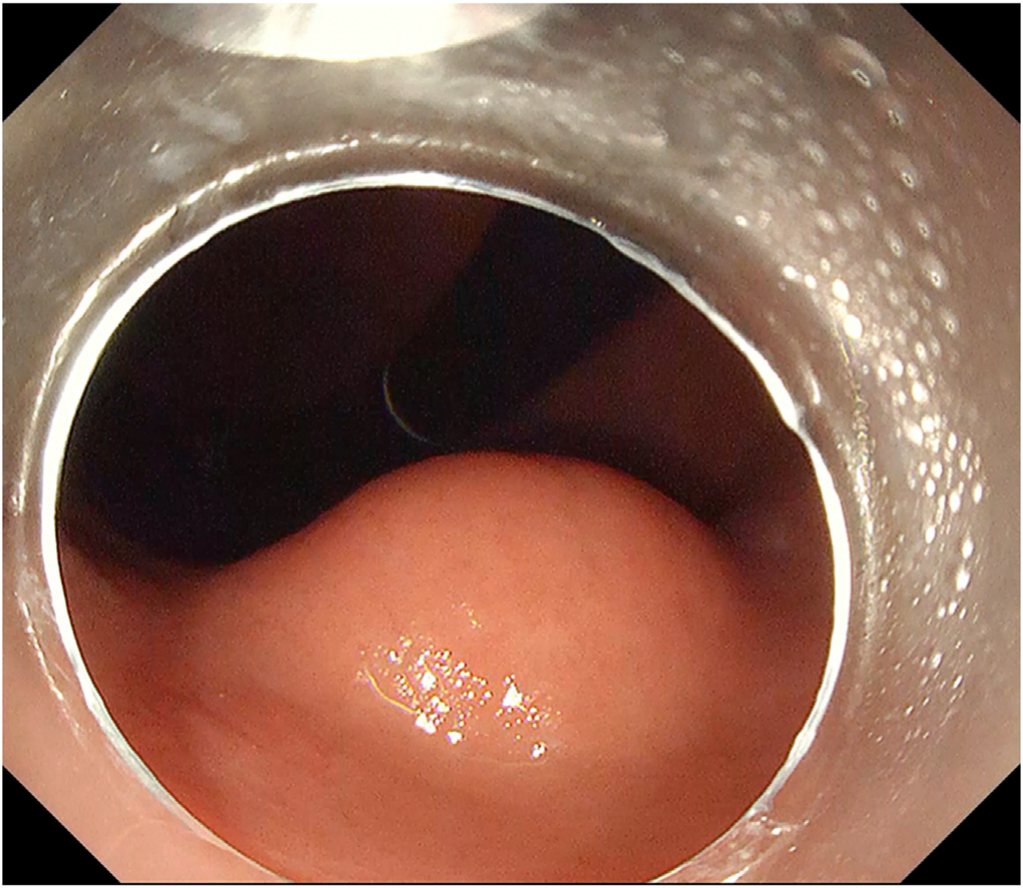
Gastric subepithelial lesion detected on screening gastroscopy. This was confirmed on EUS to have features in keeping with a GI stromal tumor.

A 47-year-old male patient was referred for screening gastroscopy at our institution. A gastric subepithelial lesion was detected in the lower body (Fig. 1). EUS confirmed a 20-mm hypoechoic lesion arising from the muscularis propria, which was in line with a GIST. The option of EFTR was discussed with the patient, and he was keen for resection. EFTR was performed and en bloc resection was achieved. Final histology showed R0 resection and confirmed that the lesion was a benign GIST.

## Equipment Used

The accessories used for dissection, such as the distal attachment, electrocautery devices, and clip-line traction, are the same in ESD and EFTR. Examples of electrocautery devices in EFTR include the Flushknife BT-S, DK2620J-B20S (Fujifilm, Tokyo, Japan) and ITknife2, KD-611L (Olympus, Tokyo, Japan). The choice of which ESD knife to use in EFTR will depend on the location of the lesion. A distal attachment is used for scope stabilization and to maintain the distance between the endoscope and lesion during dissection. Clip-line traction,^10^ which is used at the discretion of the endoscopist in gastric ESD, is a routine technique in EFTR.

However, a key difference between EFTR and ESD is that a multibending dual-channel endoscope is preferred to facilitate accessing lesions near the cardia or in the upper gastric body where GISTs are commonly located. Moreover, a dual-channel endoscope is useful when the purse-string method is performed for full-thickness defect closure. One working channel is used to pass the endoloop device, while the other channel accommodates reopenable hemostatic clips in the purse-string technique of closure, which we will highlight in this review. As pneumoperitoneum may be encountered during dissection of the muscularis propria, a 14-gauge cannula is made readily available for abdominal paracentesis during the procedure. A summary of the similarities and key differences in equipment used is highlighted in Video 1 (available online at www.videogie.org).

## Method of Procedure

**Figure 2:**
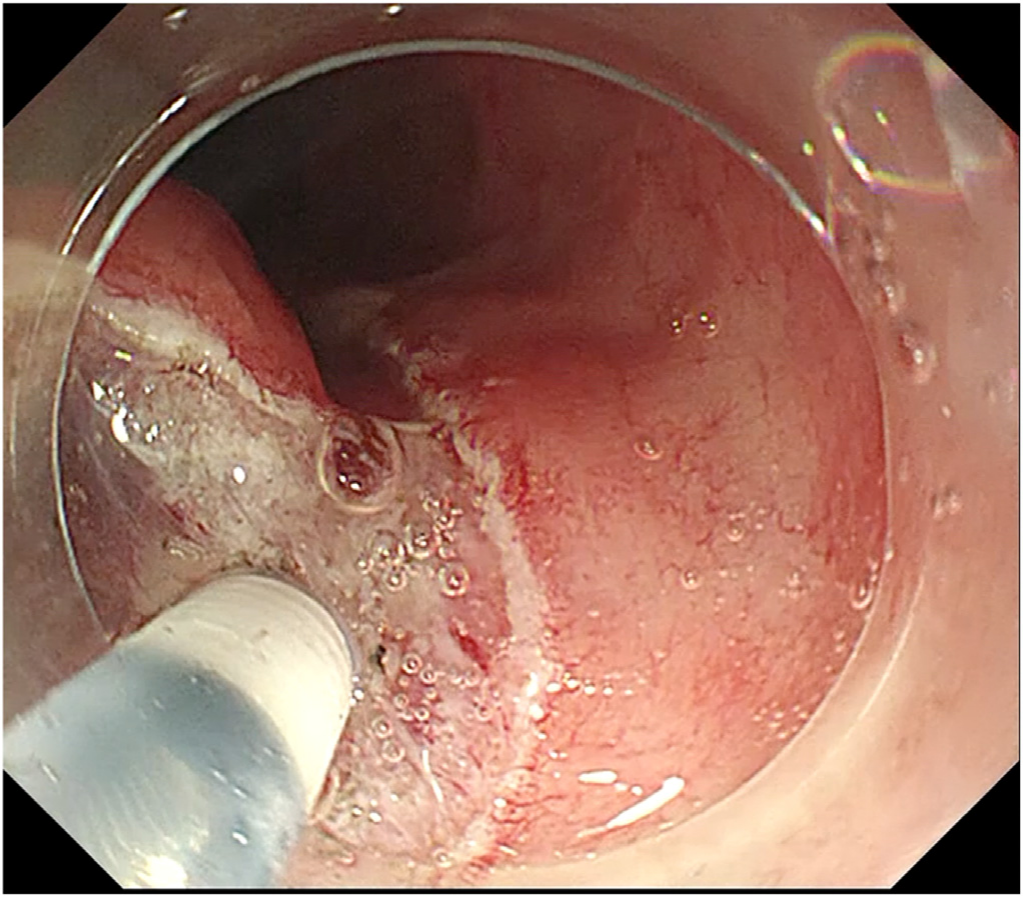
Deep mucosal incision (trimming) to muscularis propria. Note the use of a needle-type electrocautery knife for initial mucosal incision.

**Figure 3:**
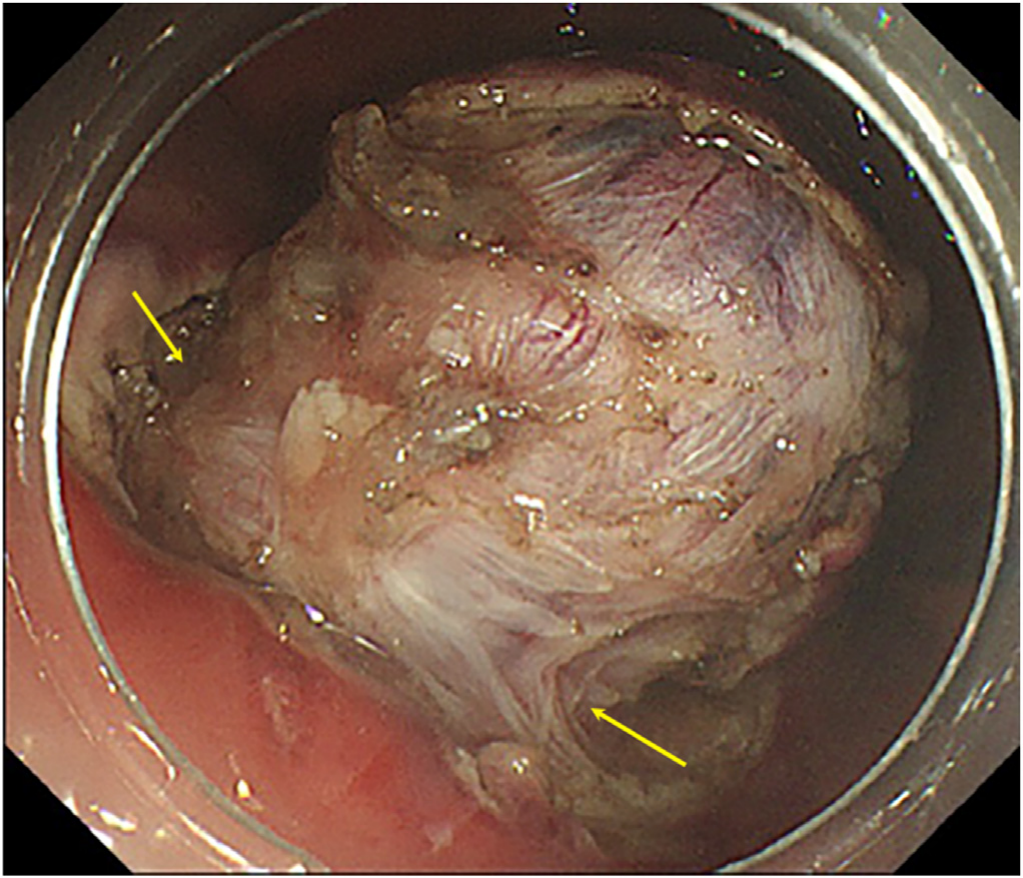
Exposure of circumferential attachment before incision of muscular layer.

Table 1 summarizes the key steps of EFTR and how they are similar to or different from ESD. During mucosal incision at the start of EFTR, a deep incision to the muscularis propria (trimming) is important (Fig. 2, Video 1). Trimming enables the most lateral submucosal fibers to be recognized and dissected easily as submucosal dissection proceeds along the muscularis propria and the surface of the tumor capsule, which in turn exposes the whole circumferential muscle attachment prior to muscular incision (Fig. 3, Video 1). The deep incision also enables the endoscopist to recognize and maintain a constant distance between the dissection plane and the surface of the tumor, preventing inadvertent injury to the tumor capsule. This can also facilitate the incision of the lateral and remnant muscle attachment toward the end of the procedure.

**Table 1:** Similarities and differences in the key steps of endoscopic full-thickness resection compared with endoscopic submucosal dissection

Similarities with endoscopic submucosal dissection	Differences
Use of electrocautery knives	Requires general anaesthesia
Importance of deep incision (trimming)	Starting mucosal incision as close to the tumor margin as possible
Traction device increases efficacy	Tissue dissection near tumor surface
Importance of vessel recognition during tissue dissection	Strong closure method for muscle defect
Paracentesis for pneumoperitoneum

**Figure 4:**
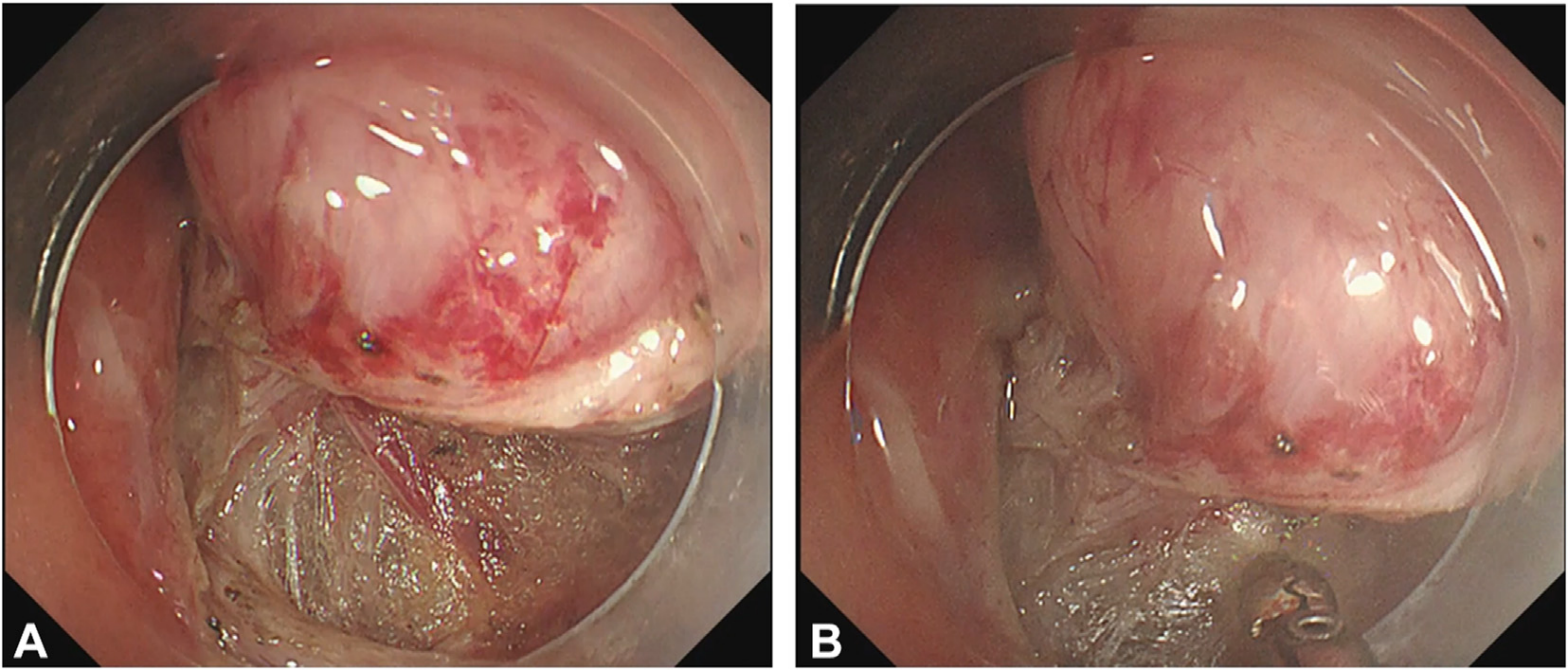
Recognition and prophylactic coagulation of visible vessels in endoscopic full-thickness resection. **A,** Recognition of vessel on serosal surface during dissection of muscle layer. **B,** Prophylactic coagulation of visible vessel to prevent intraprocedural bleeding.

In the case highlighted in the video, the lesion was located in the lesser curve of the upper body in the stomach. Initial mucosal incision and trimming were performed using a Flushknife BT-S, DK2620J-B20S, with the endoscope in the retroflexed position for controlled dissection and constant visualization of the tumor capsule. When the planes of the tumor capsule and muscularis propria were exposed, we switched to an ITknife2, KD-611L, for more efficient dissection of the circumferential muscle attachment. In a stepwise approach to EFTR like in ESD, vessels can be recognized during submucosal or muscular dissection (Fig. 4A, Video 1), and prophylactic coagulation is applied to prevent intraprocedural bleeding (Fig. 4B, Video 1).

In addition, the use of clip-line traction increases the efficiency of the procedure by exposing the muscle and serosal surfaces.^10^ In the example given in the video, the clip was deployed to the anal end of the lesion to facilitate dissection, which was performed in a retroflexed position.

Despite the similarities with ESD, there are several technical aspects in EFTR that are different. For instance, while most cases of gastric ESD can be safely performed with the patient under conscious sedation, EFTR requires general anesthesia, preferably in an operating room. In our experience of conducting procedures in an endoscopy room with the patient under deep sedation,^11^ patients often became restless after full-thickness incision was performed, which increased the risks of EFTR. Performing EFTR in an operating room with the patient under general anesthesia ensures that the patient remains stable for the duration of the procedure and enables prompt surgical intervention in the event of adverse events or when closure of the full-thickness defect was not possible.

Furthermore, while the initial mucosal incision is made approximately 5 mm outside the demarcation line of the tumor in gastric ESD, in EFTR, the initial mucosal incision is made on the edge or at the center of the tumor. This is because the mucosa shrinks after incision, which widens the size of the mucosal defect, causing defect closure to be more difficult. In EFTR, where the muscle layer is incised, the muscle defect further compounds the difficulty of closing a large mucosal defect. Thus, it is recommended that the mucosal defect be as small as possible in EFTR for a gastric GIST.

**Figure 5:**
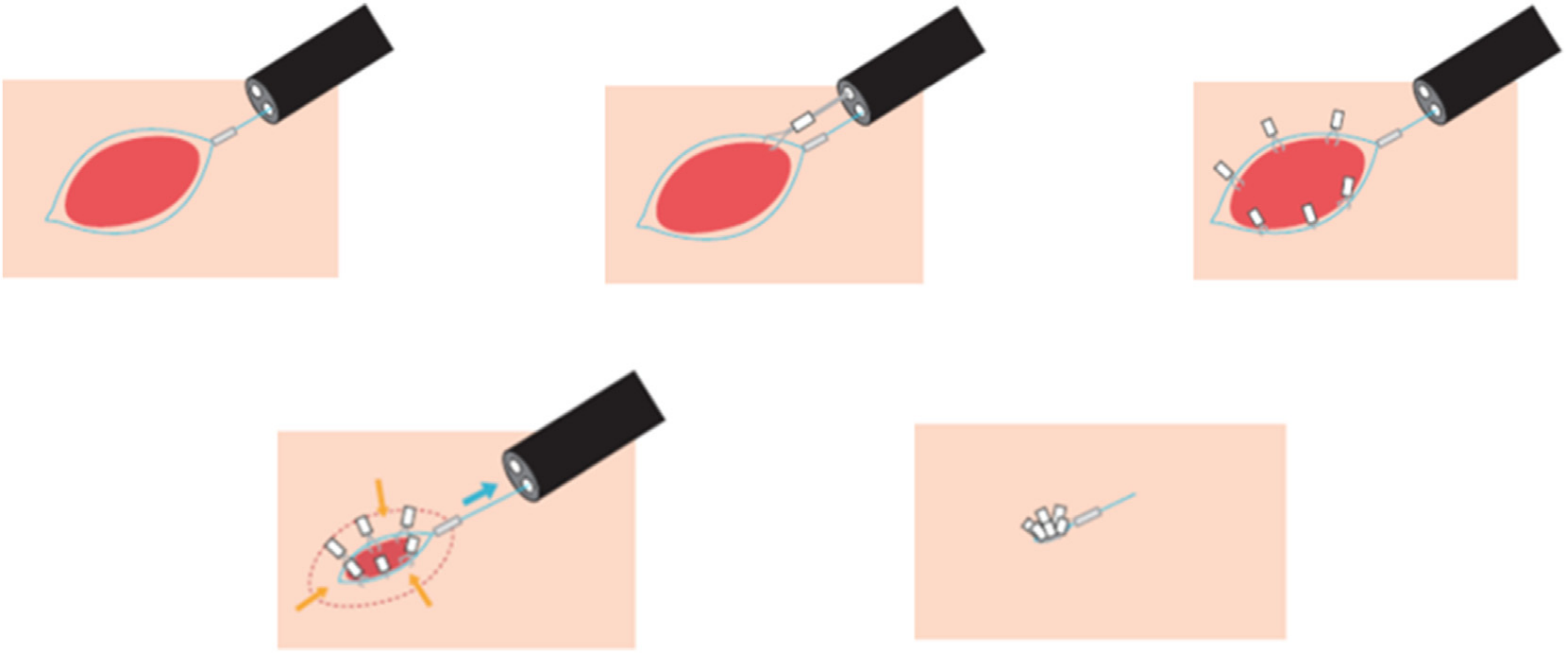
Schematic diagram illustrating the purse-string method for muscle defect closure.^11^

**Figure 6:**
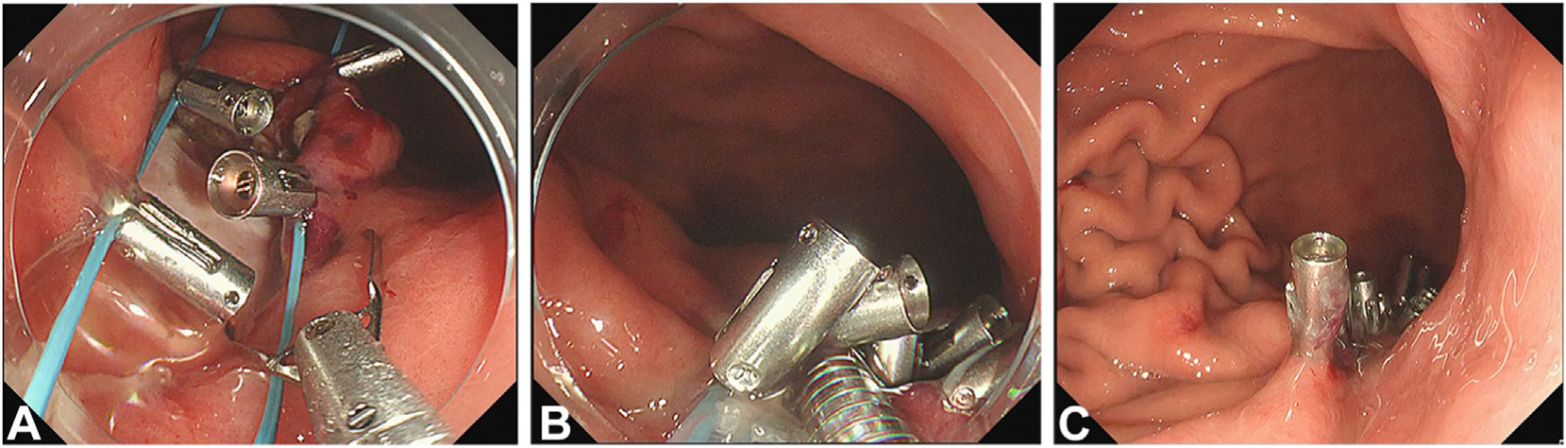
Steps in purse-string closure. **A,** Hemostatic clips are used to secure the endo-loop device along the circumference of the muscle defect. **B,** The clip sheath is used to invert stocks as the endo-loop device is closed slowly. **C,** Additional hemostatic clips are applied to the muscle defect as required.

Unlike in ESD where defect closure is not routinely performed, a strong method for closure of the muscle defect is necessary in EFTR, which is in essence a true perforation. We illustrate the purse-string method for defect closure in EFTR with a schematic diagram^11^ in Figure 5. This endoscopic suturing technique requires a dual-channel endoscope. First, an endo-loop device is passed through one of the working channels and opened in the gastric lumen close to the muscle defect. Hemostatic clips are then passed sequentially through the other working channel to secure the endo-loop device to the circumference of the muscle defect. This process usually requires 6 to 8 hemostatic clips (Fig. 6A, Video 1). After application of the last hemoclip, the clip sheath is maintained in the working channel to carefully invert the stocks of the hemostatic clips into the gastric lumen as the endo-loop device is closed slowly to completely appose the edges of the muscle defect (Fig. 6B, Video 1). Finally, additional hemostatic clips are applied as required to secure the muscle defect closure (Fig. 6C, Video 1).

## Conclusion

ESD techniques and equipment can be adapted for use in EFTR of gastric GISTs. Principles such as deep mucosal incision, the appropriate use of traction, and identification of vessels for prophylactic coagulation help to ensure a safe and efficient procedure. The main difference in EFTR is the need for general anesthesia, starting the mucosal incision inside the tumor margin, submucosal dissection around the surface of the tumor capsule, and a strong closure method for the muscle defect. Understanding these similarities and differences facilitates the implementation of EFTR for gastric GISTs by endoscopists who are familiar with ESD techniques.

## Disclosure

Dr Uedo has received honoraria for lecturing from Fujifilm Co Ltd, Boston Scientific Japan, Daiichi-Sankyo Co Ltd, Takeda Pharmaceutical Co Ltd, EA Pharma Co Ltd, Otsuka Pharmaceutical Co Ltd, AstraZeneca Co Ltd, Miyarisan Pharmaceutical Co Ltd, and AI Medical Service Inc. Dr Li has received honoraria for lecturing from Fujifilm Co Ltd and Boston Scientific. Dr Shichijo has received honoraria for lecturing from Olympus Co Ltd, EA Pharma Co Ltd, AstraZeneca Co Ltd, AI Medical Service Inc, and Janssen Pharmaceutical. Funding for this work was provided by the 10.13039/501100005150Osaka Foundation for the Prevention of Cancer and Lifestyle-related Diseases.

## Supplementary data

Video 1 Adaptation of endoscopic submucosal dissection techniques for endoscopic full-thickness resection.
